# Leaf metabolite profile of the Brazilian resurrection plant *Barbacenia purpurea* Hook. (Velloziaceae) shows two time-dependent responses during desiccation and recovering

**DOI:** 10.3389/fpls.2014.00096

**Published:** 2014-03-14

**Authors:** Vanessa F. Suguiyama, Emerson A. Silva, Sergio T. Meirelles, Danilo C. Centeno, Marcia R. Braga

**Affiliations:** ^1^Núcleo de Pesquisa em Fisiologia e Bioquímica, Instituto de BotânicaSão Paulo, Brazil; ^2^Departamento de Ecologia, Universidade de São PauloSão Paulo, Brazil; ^3^Centro de Ciências Naturais e Humanas, Universidade Federal do ABCSão Bernardo do Campo, Brazil

**Keywords:** rock outcrops, drought, metabolomics, dehydration tolerance, caffeoyl-quinic acids, raffinose-family oligosaccharides

## Abstract

*Barbacenia purpurea* is a resurrection species endemic to rock outcrops, in Rio de Janeiro, Brazil. It tolerates great temperature variations, which are associated to periods of up to 30 days without precipitation. Using a metabolomic approach, we analyzed, under winter and summer conditions, changes in the leaf metabolite profile (MP) of potted plants of *B. purpurea* submitted to daily watered and water deficit for at least 20 days and subsequent slow rehydration for 5 days. Leaves were collected at different time points and had their MP analyzed by GC/MS, HPAEC, and UHPLC techniques, allowing the identification of more than 60 different compounds, including organic and amino acids, sugars, and polyols, among others. In the winter experiment, results suggest the presence of two time-dependent responses in *B. purpurea* under water stress. The first one starts with the increase in the content of caffeoyl-quinic acids, substances with strong antioxidant activity, until the 16th day of water suppression. When RWC reached less than 80 and 70%, in winter and summer respectively, it was observed an increase in polyols and monosaccharides, followed by an increment in the content of RFO, suggesting osmotic adjustment. Amino acids, such as GABA and asparagine, also increased due to 16 days of water suppression. During rehydration, the levels of the mentioned compounds became similar to those found at the beginning of the experiment and when compared to daily watered plants. We conclude that the tolerance of *B. purpurea* to dehydration involves the perception of water deficit intensity, which seems to result in different strategies to overcome the gradient of water availability imposed along a certain period of stress mainly during winter. Data from summer experiment indicate that the metabolism of *B. pupurea* was already primed for drought stress. The accumulation of phenolics in summer seemed to be more temperature and irradiance-dependent than on the RWC.

## Introduction

Rock outcrops are usually found in distinct Brazilian biomes, some of them located within biodiversity hotspots of global importance, such as the Atlantic rain forest of southeastern Brazil (Myers et al., 2000; Scarano, 2007). The rock outcrop vegetation bears a peculiar flora, characterized by high biodiversity and degree of endemism. Similarities have been found among the South American and African rock-outcrop communities. However, dissimilar patterns of plant surface occupation are observed among them, indicating the existence of local environmental peculiarities affecting the community performance. In the Rio de Janeiro state, monocotyledons are the most frequent vascular plants found in these sites, with the predominance of species belonging to Bromeliaceae and Velloziaceae (Meirelles et al., 1999).

The stressful habitat of rock outcrops requires particular plant adaptations to survive to their limiting conditions such as low temperatures, drought, and soil scarcity. Some vascular plants from Brazilian rock outcrops have been described as desiccation tolerant species but studies with them are still scarce (Meguro et al., 1977; Garcia, 1997; Meirelles et al., 1997; Aidar et al., 2010). Desiccation tolerance is the ability of an organism to deal with extreme water deficit surviving the loss of more than 95% of its cellular water, remained viable for long periods in a state of anabiosis and return to its normal cellular metabolism after water becomes available (Farrant et al., 2007, 2009; Morse et al., 2011; Dinakar et al., 2012). Although it is commonly found in seeds and pollen grains, which can withstand air dryness for certain periods of time, only a small group of vascular angiosperms termed “resurrection plants” has also evolved desiccation tolerance and can revive their vegetative tissues from an air-dried state (Bartels, 2005). The desiccation tolerance in these plants is related to peculiar protection mechanisms, which include the presence of complex metabolic machinery associated with dehydration and rehydration processes (Morse et al., 2011; Gechev et al., 2012).

Changes in levels of carbohydrates and other protective molecules have been widely reported on most of the studied resurrection plant species as a common response to dehydration (Farrant et al., 2009). Besides sustaining growth and regulating gene expression, sugars have been implicated in the cell osmotic adjustment, stabilization of membrane structures and glass state formation during desiccation, and in providing carbon skeletons for recovering during the rehydration process (Vertucci and Farrant, 1995; Hoekstra et al., 1997; Oliver et al., 1998; Farrant et al., 2007, 2009; Peters et al., 2007). Additionally, carbohydrates have also been linked to the protection against oxidative stress, acting as antioxidants with ROS-scavenging capacity (Van den Ende and Valluru, 2009). The rapid capacity to accumulate sucrose and raffinose-family oligosaccharides (RFO) has been described in dehydrating resurrection angiosperms in response to dehydration (Morse et al., 2011). More than direct products of photosynthesis, these sugars seem to accumulate as result of the massive conversion of carbohydrate reserves such as starch or, as reported for *Craterostigma* spp., the uncommon sugar 2-octulose (Bianchi et al., 1991; Dinakar et al., 2012).

In contrast to carbohydrates, few studies have examined the contribution of secondary metabolite changes as protective mechanisms related to tolerance desiccation in resurrection plants. In a study performed with dry leaves of the African resurrection species *Myrothamus flabellifolia*, high levels of 3,4,5-tri-*O*-galloylquinic acid, which acts as a potent antioxidant, were found in the vacuole (Moore et al., 2005). Although the amount of total polyphenols varied among the resurrection plants, the content detected in leaves of *M. flabellifolia* was much higher than that found in other desiccation tolerant species (Farrant et al., 2007). In drying leaves of *Boea hygrometrica*, a proteomic approach showed upregulation of proteins related to phenolic metabolism (Jiang et al., 2007). Gechev et al. (2013) showed, from integrated transcriptome and metabolome approaches, that the resurrection plant *Haberlea rhodopensis* has both inducible and constitutive mechanisms for tolerating water deficit, including the increase in several amino acids and secondary metabolites.

Although considerable progress has been made in understanding the desiccation tolerance in resurrection plants (Morse et al., 2011; Dinakar et al., 2012) there are different aspects and peculiarities, which have still to be explored. The study of species-specific responses can contribute to reveal hidden mechanisms allowing resurrection plants to survive under stressful conditions. Metabolite profiling is a useful and important tool to identify global metabolites associated with plant response to stresses. However, comprehensive metabolomic approaches have been conducted only recently and with few resurrection species as *Sporolobus stapfianus* and *H. rhodopensis* (Oliver et al., 2011; Gechev et al., 2013). A limiting metabolomic study was first performed on *Mohria caffrorum* comparing sugar profiles between desiccation-tolerant and sensitive plants (Farrant et al., 2009).

Despite the report of about 30 potential desiccation tolerant vascular plants in southeastern Brazil (Meirelles et al., 1997) very little is known about their physiology and biochemistry in desiccated and rehydrated conditions and their metabolic changes in response to drought. A first physiological study was performed with *Barbacenia purpurea* (= *Pleurostima pupurea*, Velloziacae), which occurs in monocotyledons mats on soil islands, some meters above the tidal zone, in the Pão de Açúcar rock outcrop in Rio de Janeiro, southeastern Brazil, where it is exposed to great temperature variations, frequent periods without precipitation and to salinity effects (Meirelles et al., 1999; Aidar et al., 2010). Although Aidar et al. (2010) have shown the presence of some strategies to deal with extreme water loss in *B. purpurea*, the biochemical responses implicated on desiccation tolerance as well as the effects of seasonality on the species performance are unknown.

To extend our understanding of the responses of Brazilian desiccation tolerant species to drought, we assessed, using metabolomic analysis, changes in primary and secondary metabolism of *B. purpurea* associated with its physiological responses to dehydration and subsequent rehydration and whether these changes are influenced by season.

## Materials and methods

### Plant material

**Figure 1:**
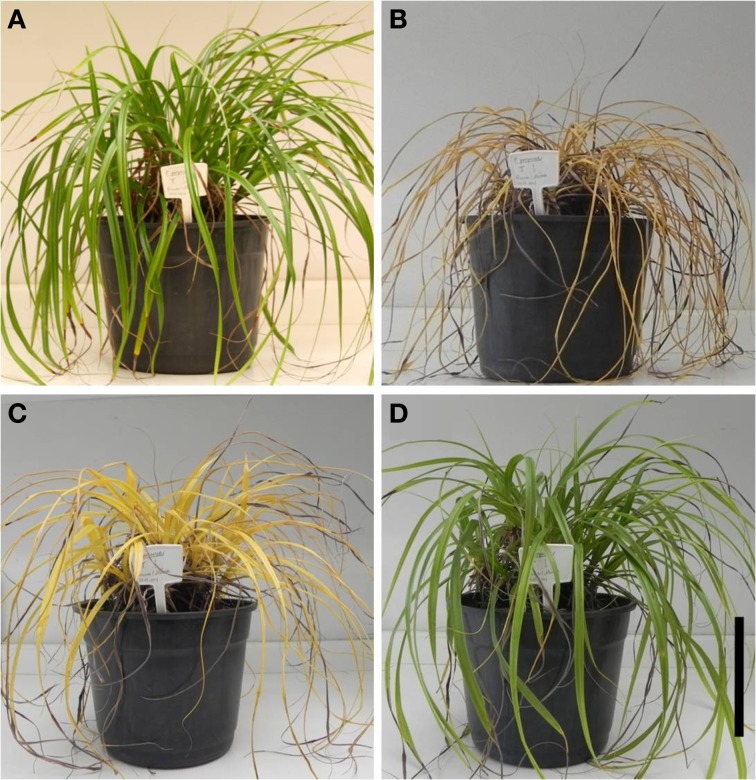
**General appearance of the plants of *Barbacenia purpurea* along the dehydration and rehydration**. Aspect of the daily watered plants **(A)** and plants under water suppression **(B)** and rehydration **(C,D)** during the winter season. Plants of 0 days **(A)** and 20 days **(B)** of dehydration and 12 h **(C)** and 38 h **(D)** after rehydration. Scale bar = 15 cm.

**Figure 2:**
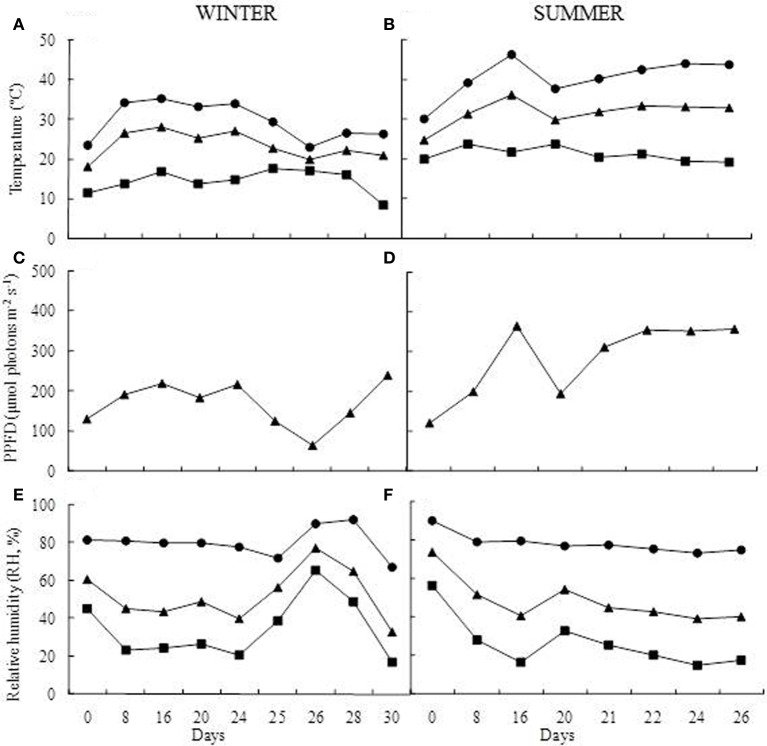
**Greenhouse growth conditions**. Temperature (°C) **(A,B)**, photosynthetic photon flux density (PPFD, μmol photons m^−2^ s^−1^) **(C,D)**, and relative humidity (RH, %) **(E,F)**. Maximum (●), mean (▲), and minimum values (■) during of winter and summer seasons.

Adult plants of *Barbacenia purpurea* Hook. (Velloziaceae) [synonymy *Pleurostima purpurea* (Hook.) Raf.], obtained from seeds collected in a natural population from Morro da Urca near Pão de Açúcar (Sugar Loaf Montain) in Rio de Janeiro, Brazil, were maintained in pots of 5 liters containing commercial substrate (Plantmax®) inside a greenhouse under natural lighting and monitored environmental conditions (Figures 1A, 2). The pots received nutrient solution (Hoagland and Arnon, 1950) 20 days before the experiments and were daily watered until the beginning of the experiments. Six potted plants with same age and containing up to 18 apexes were used in the experiments.

### Dehydration and rehydration

For the drought imposition, plants were divided into control (C) and treated (T) groups. The control group (3 pots) was daily watered at field capacity throughout the experimental period. The treated group (3 pots) was submitted to dehydration by total water withholding, following rewatering until complete plant rehydration. The experiments were accomplished in two seasons, winter (July 2011) when water deficit was imposed for 24 days and summer (February 2012) when water deprivation was maintained for 20 days. During the experiments, the air temperature (°C), relative humidity (RH, %) and the photosynthetic photon flux density (PPFD, μmol photons m^−2^ s^−1^) were monitored inside the greenhouse using a temperature/humidity sensor (Li-1400-140, Li-Cor-Nebraska, USA) and a quantum sensor (Li- 190SA, Li-Cor—Nebraska, USA) respectively (Figure 2). The sensors were connected to a datalogger (Li-1400; Li-Cor-Nebraska, USA), which was configured to perform the measurements at intervals of 10 min, calculating daily means for each parameter evaluated.

Leaves were collected on 0, 8, 16, 20, 24 days of dehydration and after 12, 36, 84, and 132 h of rehydration, according to previous work (Aidar et al., 2010). For biochemical tests, leaves were collected into liquid nitrogen, brought to powder, and lyophilized. Six biological replicates of whole leaves were used for each analysis. All collections of plant material were carried out between 06:00 and 07:30 am.

### Evaluation of dehydration

For determination of the relative water content (RWC), leaves were collected and immediately weighted for fresh weight (FW) and then submerged in water by 24 h for obtainment of the turgid weight (TW) and placed in oven at 60°C by 72 h for dry weight (DW). The RWC was calculated according to Weatherley (1950), using the formula: RWC (%) = (FW-DW/TW-DW) × 100.

### Photosynthetic pigments content

The extraction and quantification of chlorophylls *a*, *b* and carotenoids were carried out according Hendry and Price (1993). The leaf samples were ground in a mortar with 80% acetone in a darkened room and the supernatant analyzed by spectrophotometer (SP-22, Biospectro). Six replicates of 5 cm of the median portion of leaves of each treatment were used.

### Carbohydrate extraction and analyses

The sugar extraction was accomplished with 200 mg of pooled samples (two leaves each), totaling 3 replicates for group, of lyophilized leaf material. Samples were boiled 5 min in 80% ethanol (40 mg fresh mass ml^−1^) and the supernatants were recovered by centrifugation (2000 g, 15 min). The residues were manually homogenized and re-extracted three times in boiling 80% ethanol. The supernatants were recovered by centrifugation, combined and considered as the soluble-sugar extracts. The ethanol was evaporated under vacuum and the extracts used for sugar quantification (Carvalho et al., 2013). The residues were washed with distilled water, freeze-dried and used for quantification of starch. The amounts of total carbohydrates and reducing sugars in the ethanolic extracts were determined colorimetrically by the phenol-sulphuric acid method (Dubois et al., 1956) and Somogyi-Nelson procedure (Somogyi, 1945), respectively, using glucose (Sigma Aldrich®) as standard. Aliquots of the extracts (1 ml) were deionized through an anion exchange columns Dowex (Sigma Aldrich®), using cation exchange resins 50 × 8 (100–200 mesh) and anionic 1 × 8 (52–100 mesh) and analyzed by High Performance Anion-Exchange Cromatography coupled to/Pulse Amperometric Detector (HPAEC/PAD) using an ICS 3000 Dionex system (Dionex, Thermo Scientific, USA) with a CarboPac PA-1 column (2 × 250 mm). The monosaccharides and oligosaccharides were eluted isocratically with 100 mM NaOH in flux of 0.25 ml min^−1^. The detected peaks after a run time of 35 min were compared with commercial standards.

Starch content was estimated by enzymatic analysis, using 5–10 mg of freeze-dried residue after ethanol extraction, according to Amaral et al. (2007). Enzymatic digestion was performed twice with 60 units of α-amylase from *Bacillus licheniformis* (EC 3.3.1.1; Megazyme®, Ireland) in 0.5 ml of 10 mM 3-(*N*-morpholino) propanesulfonic acid (MOPS) buffer (pH 6.0), at 75°C for 45 min, and subsequently with 15 units of amyloglucosidase from *Aspergillus niger* (EC 3.2.1.3; Megazyme®, Ireland) in 0.5 ml 100 mM sodium acetate buffer (pH 4.5) at 50°C for 30 min. Reaction was stopped by adding 100 μl of 800 mM perchloric acid and the supernatant recovered by centrifugation at 10,000 rpm in an Eppendorf® centrifuge for 2 min. Starch was determined by measuring glucose released by the enzymatic digestion after incubation with aliquots of the reagent Glucose PAP Liquiform (Centerlab®) containing glucose oxidase and peroxidase (GOD-POD), 4-aminoantipyrine and phenol. Fifty microliters of the incubation mixtures were added to 750 μ l of GOD-POD and kept at 30°C for 15 min. The absorbance was measured in an Elisa plate at 490 nm. Glucose (Sigma Aldrich®) was used as standard.

### Analysis of the metabolite profile

Aliquots of 20 mg of lyophilized leaves were extracted in 500 μl of a methanol:chloroform:water [12:5:1] solution. Fifty microliters of adonitol (0.2 mg ml^−1^) was used as internal standard and samples were agitated and centrifuged. After extraction 350 μl of water was added to 350 μl collected supernatant for polar phase separation. Three hundred microliters of the polar phase was dried under vaccum for derivatization. The samples were derivatized with pyridine, *N*-*O*-bis (trimethylsilyl) trifluoracetamide (BSTFA) and methoxyamine hydrochloride (20 mg ml^−1^ pyridine). The total ion chromatogram (TIC) and mass spectra were evaluated using the program Chem Station (Agilent) and detected peaks were identified by comparison with authentic standards and to the NIST 08 Spectral Library and confirmed by Kovats indices. The analysis by Gas Cromatography coupled to/Mass Spectrometry (GC/MS) was performed in Agilent GC 6890 series coupled to a quadrupole mass spectrometer Agilent MSD 5973N (Agilent Technologies, USA), according to the method of Roessner et al. (2001), modified, where a DB-5 ms column was used in an oven with initial temperature of 70°C by 5 min and final temperature of 280°C by 1 min, with 5°C min^−1^ increasing ratio.

### Amino acid analysis

A total of 40 μL of polar phase of the samples previously extracted for the metabolite profile (MP) analysis were used for the identification of amino acids, using a Waters Acquity Ultra Performance Liquid Cromatography (UPLC) System (Waters, Milford, USA). The material was prepared according to AccQ-Tag kit (Waters, Milford, USA) instructions. The separation occurred through an AccQ-Tag Ultra Column C18 (2.1 × 100 nm with 1.7 m) at 60°C with eluents: A—AccQ-Tag Ultra Eluent A at 10% in water, B—AccQ-Tag Ultra Eluent B at 100% (Waters), in a flow rate of 0.7 ml min^−1^. The amino acids were detected at 260 nm. The determination of the amino acids was made by comparison with commercial standards.

### Experimental design and statistical analysis

The experiments were arranged on completely randomized design with 3 or 6 replicates per treatment in each point of the analyses. Data were then analyzed by ANOVA with a posteriori comparison of the means using Tukey's test to investigate the occurrence of significant differences between treated and control plants, using the statistical package BioEstat 3.0 (Ayres et al., 2003). Significant effects were reported at P 0.05. Principal component analysis (PCA) was conducted using the values of relative abundance of each compound (obtained by different techniques) and were normalized by DW of the samples, by internal standards, when applicable, and by values obtained for the control (day 0). The PCA was performed using the software Statistica (SoftStat, Inc., Tulsa, USA). In addition, correlation between physiological and environmental parameters were evaluated using correlation analyses performed with the mean values of each parameter. Correlations were tested for significance (*P*) by Student's *t*-test.

## Results

### Dehydration and rehydration

During the experiments, air temperature, RH, and photosynthetic active radiation inside the greenhouse were in average 24°C, 52%, and 168.2 μmol photons m^2^ s^−1^ in the winter and 31°C, 79%, and 281.1 μmol photons m^2^ s^−1^in summer (Figure 2). Under induced water deficit, it was observed curling of leaf blades toward to the central nervure (Figure 1B). This curling occurred in RWC between 60 and 70% in both winter and summer experiments and was concomitant with increased leaf hardening. With rehydration the RWC increased gradually, reaching similar values of control after 84 h (RWC above 70%) in both experiments. The return of water uptake occurred gradually from leaf base toward to leaf apex (Figure 1C) and, seemingly, the return to the turgid state was observed about 90 h after rehydration for both seasons (Figure 1D).

**Figure 3:**
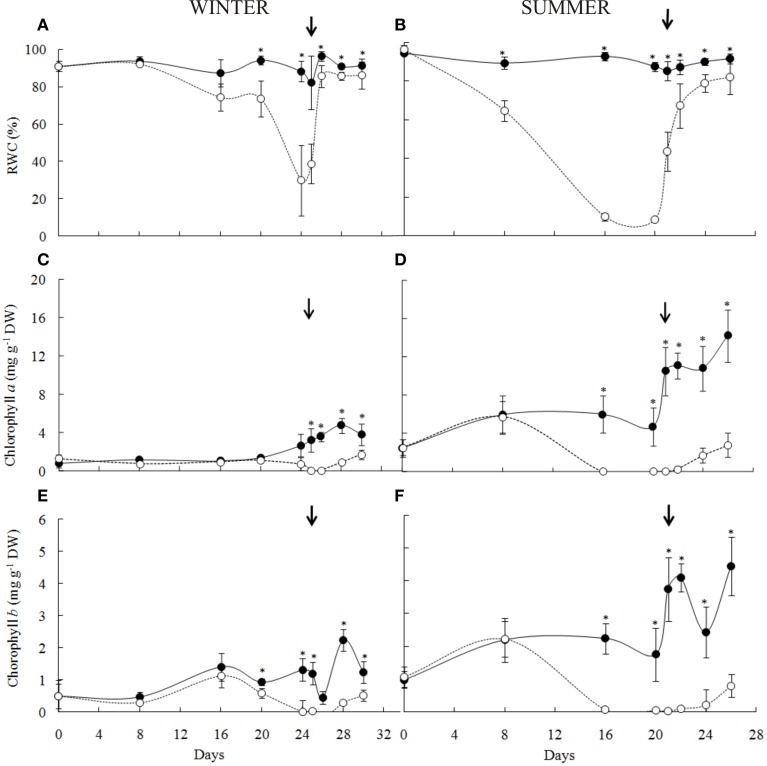
**Ecophysiological parameters of *Barbacenia purpurea* during dehydration and rehydration**. Leaf relative water content **(A,B)**, chlorophyll *a* **(C,D)** and chlorophyll *b* **(E,F)** in leaves of daily watered plants (control group—**•**) and plants under water suppression (treatment group—○) in winter (left side) and summer (right side) season. The narrow indicates the day of rehydration. ^*^Significant difference between treatments at *P* < 0.05 (*n* = 6 ± *SD*).

In the winter experiment, the leaf dehydration of *B. purpurea* started from the 16th day of the water deficit imposition reaching minimum values corresponding to 29% of RWC after 24 days (Figure 3A). In the summer, the RWC decreased quickly during the first days of treatment, reaching the minimum value of 9% between the 16th and the 20th day of water suppression (Figure 3B).

The levels of chlorophylls *a* (Figures 3C,D) and *b* (Figures 3E,F) decreased to undetectable values at RWC below 30% in both winter (24 days) and summer (16 days). The re-synthesis of chlorophylls occurred from 36 h at RWC above 80% in both experiments following the return of leaf turgidity.

### Soluble carbohydrates and starch

**Figure 4:**
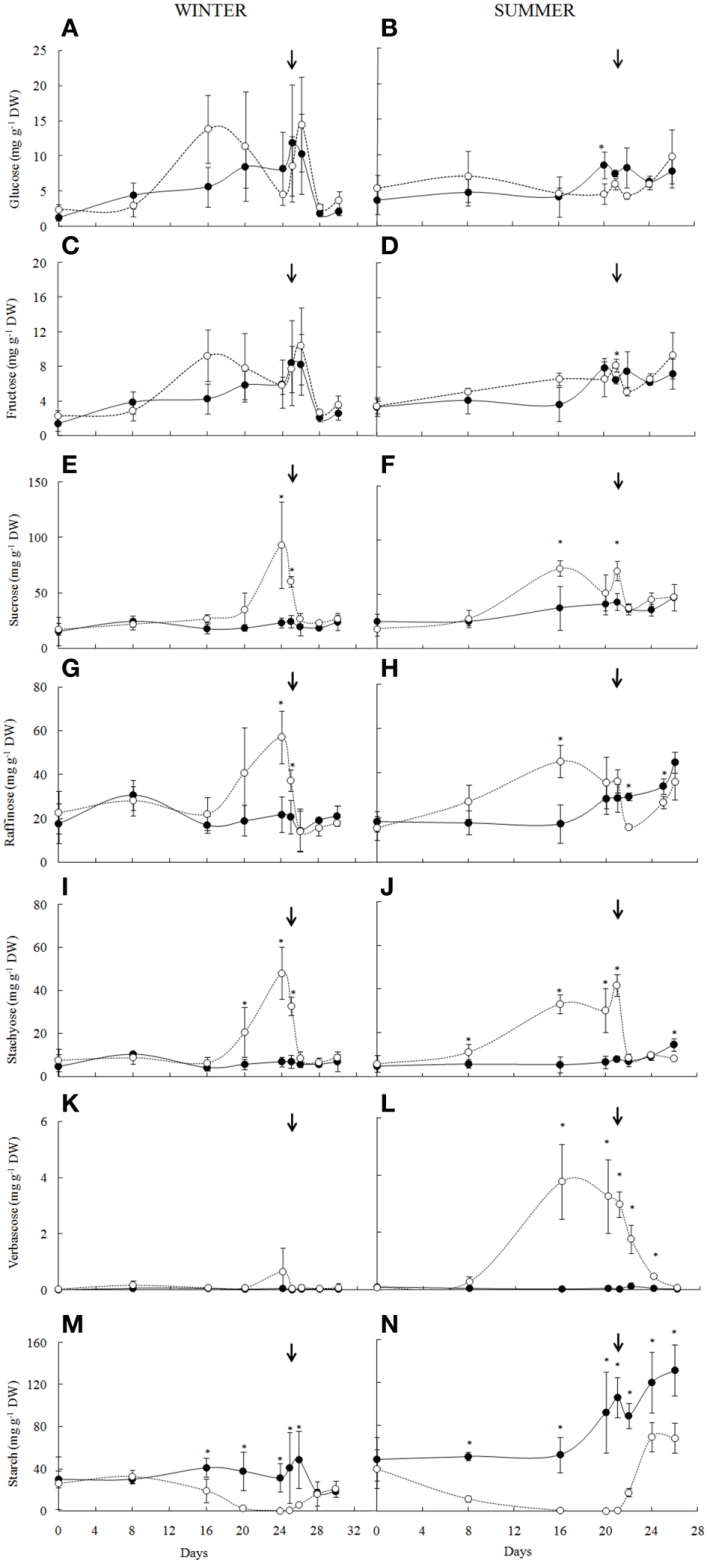
**Carbohydrates analysis of the *Barbacenia purpurea* leaves during dehydration and rehydration**. Glucose **(A,B)**, fructose **(C,D)**, sucrose **(E,F)**, raffinose **(G,H)**, stachyose **(I,J)**, verbascose **(K,L)**, and starch **(M,N)** in leaves of daily watered plants (control group—**•**) and plants under water suppression (treatment group—○) in winter (left side) and summer (right side) season. The narrow indicates the day of rehydration. ^*^Significant difference between treatments at *P* < 0.05 (*n* = 3 ± *SD*).

The soluble sugar analysis in leaves of *B. purpurea* in the winter and summer experiments allowed the quantification of glucose, fructose, sucrose and the RFO, stachyose, and verbascose (Figure 4). With water deficit imposition no significant changes were observed in glucose (Figures 4A,B) and fructose (Figures 4C,D) contents, however sucrose (Figures 4E,F) and RFO (Figures 4G–L) increased significantly. In winter, the increased levels of sucrose, raffinose, and stachyose occurred from the 20th day, in a RWC bellow 60%, while on summer those changes occurred from 8th day on (RWC around 60%), reaching a maximum value on the 16th day of water deficit when RWC was already below 20%. Sucrose and raffinose levels were reduced on the 20th day, whereas the increased content of stachyose was maintained. An increase in verbascose from the 8th was also observed. After 84 h of rehydration (RWC above 70%), the contents of sucrose, raffinose, and stachyose were similar to the control plants in both experiments, whereas verbascose, in summer, decreased slowly, following rehydration.

The starch content decreased to undetectable levels from 16th day in winter (Figure 4M) and in the first days of drought imposition in summer (Figure 4N). In both seasons the decrease started in RWC around 70%. After the rehydration the re-synthesis of starch was detected within 36 h in both experiments (RWC above 60%).

Besides differences among treatments, under water deficit conditions, high significant negative correlations were observed between RWC and sucrose (*r* = −0.958, *P* = 0.00007), raffinose (*r* = −0.821, *P* = 0.009), and stachyose (*r* = −0.948, *P* = 0.0001) in winter and sucrose (*r* = −0.716, *P* = 0.045), raffinose (*r* = −0.719, *P* = 0.044), stachyose (*r* = −0.824, *P* = 0.011), and verbascose (*r* = −0.925, *P* = 0.0009) in the summer. Starch correlates positively with RWC of water-suppressed plants in both winter (*r* = 0.749, *P* = 0.027) and summer (*r* = 0.755, *P* = 0.030) conditions.

### Metabolite profile

Under our experimental conditions, investigation of the components of the MP of leaves of *B. purpurea* allowed the identification of 67 primary and secondary metabolites, including 23 amino acids, 19 organic acids, 11 carbohydrates, 8 polyols, 3 fatty acids, besides 3 other metabolites, some of them showing changes in response to water deficit when compared to control (Supplementary material – S.1).

**Figure 5:**
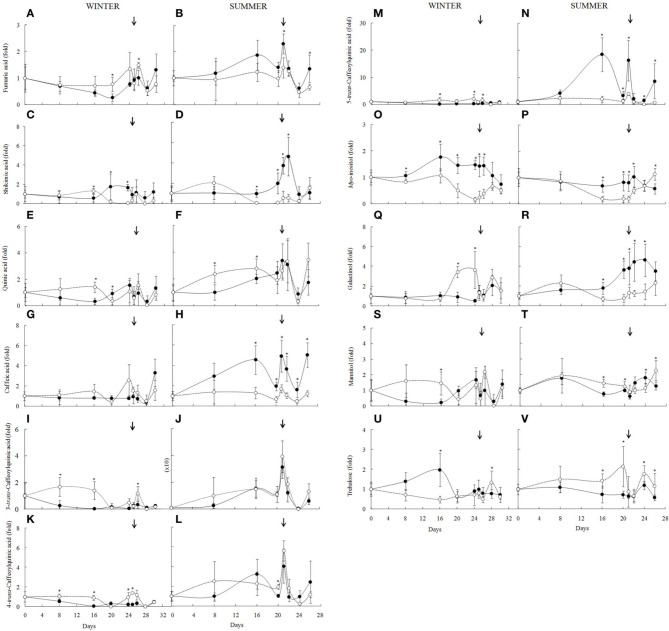
**Metabolite profile of the *Barbacenia purpurea* leaves during dehydration and rehydration**. Organics acids **(A–N)**, polyols **(O–T)**, trehalose **(U,V)** in leaves of daily watered plants (control group—**•**) and plants under water suppression (treatment group—○) in winter (left side) and summer (right side) season. The narrow indicates the day of rehydration. ^*^Significant difference between treatments at *P* < 0.05 (*n* = 6 ± *SD*). Data were normalized by the average of the values of Day 0 for each experiment.

In the winter, fumaric acid increased after the 16th day of drought, returning to the control levels after rehydration (Figure 5A), but remaining unchanged on the summer experiment (Figure 5B). Shikimic acid content also showed a tendency to increase in RWC around 70% in the first days of experiment, decreasing with intensification of water deficit in both experiments in RWC below 60%. After 12 h of rehydration (RWC above 40%) it reached the control levels (Figures 5C,D). Changes in shikimic acid correlates negatively with RWC of well-watered plants (*r* = −0.754, *P* = 0.03) only in summer.

Differences between winter and summer season were observed in the chlorogenic acids. Among them, the 3-*trans*-caffeoylquinic acid (Figure 5I) showed increased content in treated plants from the 8th to the 16th day in the winter (RWC below 90%), as well as in 5-*trans*-caffeoylquinic acid (Figure 5M), whereas the 4-*trans*-caffeoylquinic acid (Figure 5K) decreased after the 16th day (RWC around 60%). In the summer, the 3-*trans*-caffeoylquinic acid remained unchanged, however 4-*trans*-caffeoylquinic acid increased substantially after the 20th day (Figures 5J,L). On the other hand, 5-*trans*-caffeoylquinic acid decreased with water suppression after 16 days (Figure 5N). In the summer experiment the 3-*trans-caffeoylquinic* acid did not change significantly but its content was 25 times higher than that found in the winter. We also observed increased levels of quinic acid in the first 16 days of experiments (RWC between 70 and 60%) in both experiments (Figures 5E,F). This organic acid is a precursor of *trans*-caffeoylquinic acid, as well as caffeic acid, which changed in response to drought only in summer, decreasing significantly (Figures 5G,H). In fact, during summer caffeic acid in the well-watered plants correlated positively with temperature (*r* = 0.67, *P* = 0.06), irradiance (*r* = 0.67, *P* = 0.06) and negatively with RH (*r* = −0.65, *P* = 0.07). This was not observed for caffeoylquinic acids, which showed low correlation coefficients. On the other hand, a negative significant correlation between 3-*trans-caffeoylquinic* acid and temperature (*r* = −0.73, *P* = 0.03) was observed during winter in the well-watered plants.

The levels of *myo*-inositol (Figures 5O,P) also changed in response to water deficit, decreasing from the 16th day on when RWC was below 60% in winter (*r* = 0.680, *P* = 0.05) and summer (*r* = 0.868, *P* = 0.005). This decrease overlaps with significant increase in galactinol in the winter (Figure 5Q), while in summer (Figure 5R) a tendency to increase was observed after the 8th day, decreasing from the 16th day. Mannitol showed an increase of its content with 16 days of experiment (winter and summer), decreasing after 20 days of water deficit, reaching the control values after rehydration (Figures 5S,T). Trehalose showed opposite results, decreasing on winter (Figure 4U) and increasing on summer as the drought intensified (Figure 5V).

**Figure 6:**
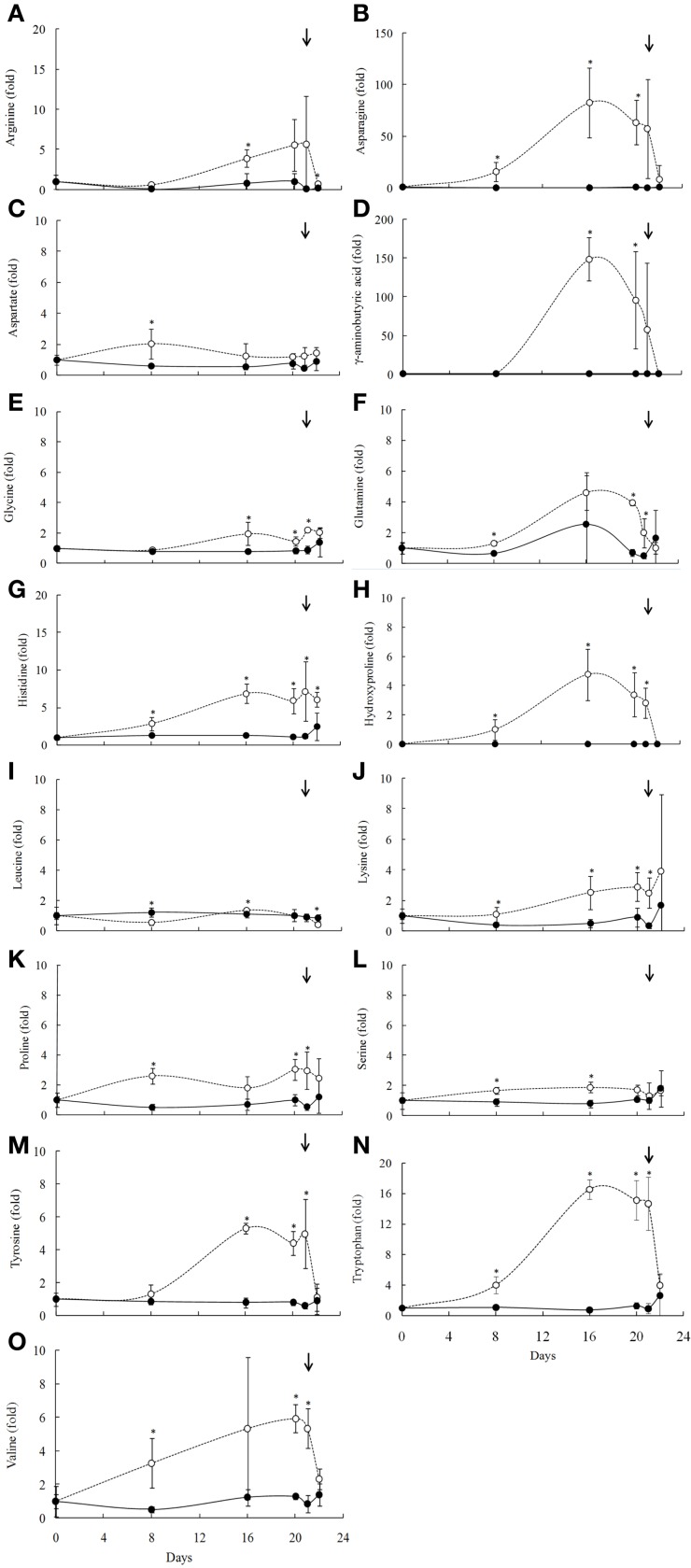
**Amino acids profile of the *Barbacenia purpurea* leaves during dehydration and rehydration (A–O)**. Relative proportion of the amino acids in leaves of daily watered plants (control group—**•**) and plants under water suppression (treatment group—○) in summer season. The narrow indicates the day of rehydration. ^*^Significant difference between treatments at *P* < 0.05 (*n* = 6 ± *SD*). Data were normalized by the average of the values of Day 0 for each experiment.

Due to a technical problem, the characterization of the amino acid composition in leaves of *B. purpurea* was taken only on summer experiments. Of the 23 detected amino acids, 16 showed changes in their levels upon water deficit (Figure 6). The treatment resulted in increased contents of aspartic and glutamic acid, glutamine, histidine, lysine, proline, serine, tryptophan, tyrosine, and valine from the 8th day of water deficit (RWC above 60%). Asparagine and gamma-amynobutyric acid (GABA) increased dramatically after 8 days of water deficit imposition (RWC above 60%) (Figures 6B,D). The amino acids arginine and glycine increased from the 16th day (RWC around 10%). Leucine decreased at 8th day of the drought (Figures 6A,E). Generally, the levels of the amino acids reached the control levels slowly after rehydration (Figure 6I).

**Figure 7:**
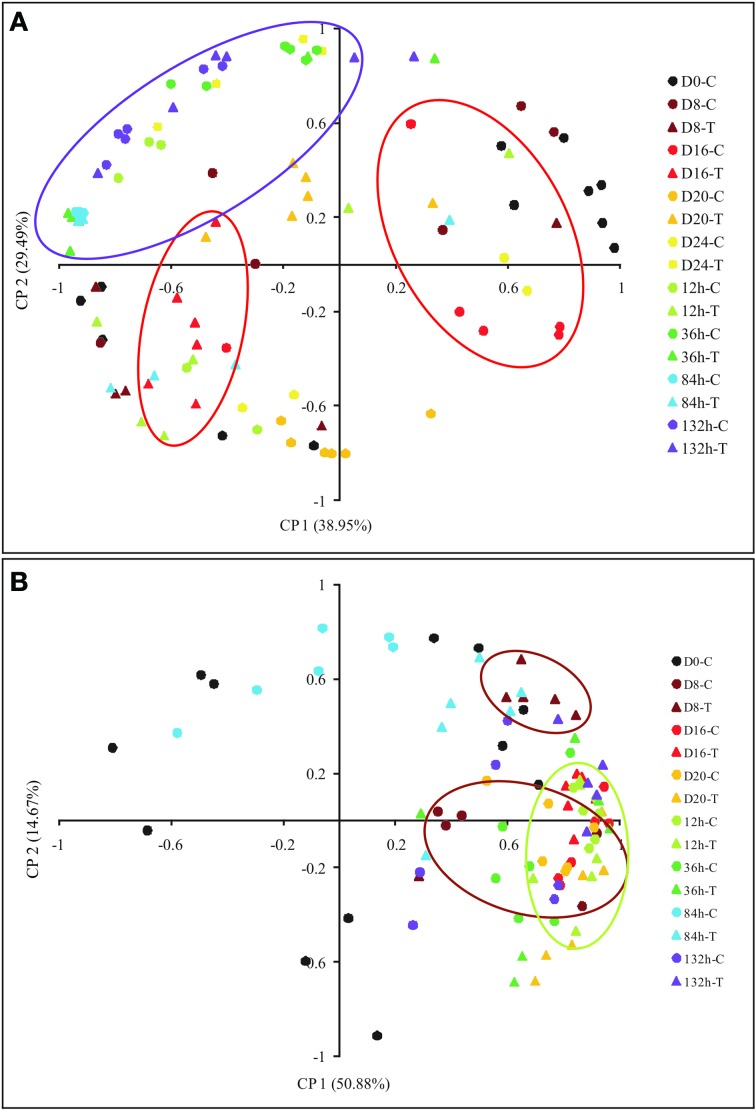
**Principal component analysis (PCA) of metabolites of *Barbacenia purpurea* during dehydration and rehydration**. PCA of metabolites quantified in leaves of daily watered plants (C) and plants under water suppression (T), during winter **(A)** and summer **(B)**. PCA is presented as the combination of the first two dimensions. Circles and triangles represent control and treated plants, respectively. Black color = Day 0, dark red = Day 8 after dehydration, light red = Day 16, orange = Day 20, yellow = Day 24 (only winter), light green = 12 h after rehydration, dark green = 32 h, blue = 84 h and purple = 132 h. Each point represents an independent biological sample. Red and purple circles represent the grouped samples at day 16 after water deficit and at 132 h after rehydration in winter, respectively, **(A)**. Brown and green circles represent the grouped samples at day 8 after water deficit and 132 h after rehydration in summer, respectively, **(B)**.

A PCA based on MP showed variations during plant dehydration, where component 1 and 2 explained more than 65% of the metabolites changes (Figure 7). In winter those changes can be clearly observed from the 16th day and start to be restored after 12 h of rehydration, reaching very similar MP after 132 h. In summer, the changes could be evaluated earlier, from the 8th day and was restored 12 h after rehydration.

## Discussion

### Dehydration

The reduction of the water content in the soil in both winter and summer experiments led to changes in the metabolism of *B. purpurea*. The biochemical responses of the *B. purpurea* were similar between seasons but variations were observed in some compounds and in their relative proportions when compared to daily watered plants. The dehydration of foliar tissues in winter occurred from 16th day and reached minimum values of RWC at 29% only after 24 days. In the summer the RWC was drastically reduced in the first days, reaching 9% on the 16th day. This difference can be explained as a result of the higher vapor gradient in the soil-plant-atmosphere in summer compared to winter, due to higher temperatures, with maximum values above 40°C (Figure 2A). Under natural environment, these plants are exposed to similar conditions of dehydration, since they grow in rock outcrops and are subject to large temperature variations and exposure to intense UV radiation.

Accompanying the dehydration, leaf curling and hardening occurred in both seasons, leading to a reduction in the leaf surface area. This is a common response in resurrection plants and has been related to the reduction of transpiration surface, facilitating temperature control and limiting the damage caused by radiation incidence (Sherwin and Farrant, 1998; Dinakar et al., 2012). The transpiration decrease due to the reduction of the stomatal conductance was observed in plants of *B. pupurea* (= *Pleurostima purpurea*) upon water deficit (Aidar et al., 2010). Metabolites such as fumarate have been reported as important stomatal controlers (Nunes-Nesi et al., 2007), avoiding water loss and gas exchange. Therefore, increase of fumarate in the winter experiment (Figure 5A) is in agreement with the mechanism of stomatal closure, diminishing transpiration and photosynthetic rate, as previously observed in *B. purpurea* by Aidar et al. (2010).

In response to water deficit and in RWC lower than 40% occurred chlorophyll degradation in *B. pupurea*. This behavior evidences the poikilochlorophyllous strategy, which is a characteristic of monocots (Farrant, 2000; Porembski and Barthlott, 2000). As seen in a previous study (Aidar et al., 2010), *B. purpurea* degrades the components of the photosynthetic apparatus only at the final stage of drying. The degradation of chlorophylls possibly occurs to prevent excessive absorption of UV radiation, as reported for *Xerophyta viscosa* (Sherwin and Farrant, 1998).

Concerning the carbohydrates, the increase of sucrose content is widely observed among resurrection plants under water deprivation (Albini et al., 1999; Illing et al., 2005; Farrant et al., 2007, 2009; Peters et al., 2007; Toldi et al., 2009). By inducing desiccation on *B. purpurea*, we observed expressive increase on sucrose levels. Unlike observed by Farrant et al. (2009) for *Mohria caffrorum*, in *B. purpurea* glucose and fructose did not have their levels decreased, suggesting that other carbon source than these monosaccharides was utilized by the plant to synthesize sucrose. Our results suggest that the increase in sucrose was favored by availability of carbon skeletons from starch mobilization, which decreased in both seasons upon reduction of water availability, as also observed in *Trifolium repens* (Lee et al., 2008) and *H. rhodopensis* (Gechev et al., 2012). The increase in sucrose is a characteristic of the last steps in cell protection for anabiosis (reviewed by Toldi et al., 2009), and occurs normally at a RWC below 60%, or in some cases below 20%, indicating that the increase in carbohydrates in these plants is in the final stage of drying. In fact, the highest correlation coefficients were found between RWC and sugars. These correlations reinforced the influence of the water status on carbohydrate levels as a late response to water deficit in *B. purpurea* in summer and winter conditions.

In the winter, we observed a decrease in the levels of *myo*-inositol and increase of galactinol and sucrose, which are precursors of the synthetic route of the raffinose family oligosaccharides (RFO) (Peterbauer and Richter, 2001), indicating that *myo*-inositol was utilized for synthesis of their related compounds. As observed for other sugars, these changes were significantly correlated with RWC. Our results are in agreement with those previously reported to the resurrection plant *Boea hygroscopica*, in which galactinol was proposed to act as donor of galactosyl for synthesis of RFO under water restriction (Albini et al., 1999). Accumulation of RFO in considerable concentrations in response to water deficit in resurrection plants was first reported to *X. viscosa*. Increased amounts of sucrose, raffinose, stachyose, and verbascose were detected under dried conditions, decreasing as the turgor evolved with rehydration (Peters et al., 2007). In *B. purpurea*, a significant increase in RFO was observed in RWC below 60%, whereas verbascose was synthesized mainly in the summer. In *H. rhodopensis*, Gechev et al. (2013) demonstrated that the accumulation of raffinose and verbascose was coincident with a strong induction of stachyose synthase in water-deficient samples, suggesting the involvement of RFO in the acquisition of tolerance against desiccation. Although no enzyme activity measurements were done in the present work, the earlier and higher accumulation of verbascose in *B. pupurea* during summer when compared to winter can be interpreted as a result of an increase in the activity of stachyose synthase when RWC decreased to 60%. Although the role of RFO in desiccation tolerance is not yet clear, it has been hypothesized that they act on cell stabilization during drying, interacting with the phosphate groups bound to lipids of membranes and with macromolecules, altering the fluidity of cytoplasm in a process characterized by reversible cell vitrification, in addition to regulating the osmotic adjustment (Hoekstra et al., 1997; Farrant et al., 2012). These sugars have been also suggested as possible source of carbon for sucrose synthesis in *Craterostigma plantagineum* (Norwood et al., 2003).

As previously reported to *Myrothamus flabelifolia*, in leaves of *B. pupurea* a small increase in the levels of trehalose was also observed with drying, but only in the summer experiment. Trehalose is a non-reducing sugar that is thought to act as a protective compound against water removal during freezing or dehydration, but the precise function of this disaccharide in plants remains unclear (Klerk and Pumisutapon, 2008; Mollo et al., 2011). With few exceptions, its presence is barely detectable during dehydration in most tested plants (resurrection or not) indicating that it probably does not act as protective osmolyte (Lunn, 2007). Trehalose-metabolism genes are widespread in all plant groups and overexpression of a gene for trehalase in *A. thaliana* led to improved survival during abiotic stresses (Lunn, 2007; Van Hutte et al., 2013). Recently it was demonstrated that the metabolism of trehalose is implicated in stomata closure mediated by ABA (Gómez et al., 2010; Vandesteene et al., 2012). Therefore, a light increase in trehalose level may not contribute to cell osmotic adjustment but changes in its content as observed in *B. pupurea* could modulate responses leading to the induction of drought avoidance mechanisms at the beginning of the water deficit imposition.

Secondary metabolism develops also a crucial role for the survivor through a stressful environment in resurrection plants. The performance of derivates of quinic acid, for example, has been reported by Moore et al. (2005), who observed the presence of 3,4,5-tri-*O*-galloylquinic acid in *M. flabelifolius* and found that this acid has a role against deleterious effects of reactive oxygen species (ROS) and could, therefore, still be active in the protection of cell membranes against damage caused by desiccation. Differently of *M. flabelifolius*, we found in *B. pupurea* caffeoylquinic acid devivatives. The chlorogenic acids are known by its activity against a potential oxidative damage caused by ROS (De Maria and Moreira, 2004; Jaiswal et al., 2011). In *B. purpurea*, the presence of the derivates of chlorogenic acids in larger quantities in the summer suggests that these acids are present in major quantities during greater radiation availability and higher temperatures, indicating an early response by *B. purpurea* to combat a possible oxidative damage. In fact, our data suggest that the over accumulation of phenolics during summer is more temperature and irradiance-dependent than to the water content. As expected, high and significant correlation between temperature and irradiance was observed (*r* = 0.89, *P* = 0.003). During winter, when temperature and light intensity are lower, we observed an inverse correlation, which indicates that accumulation of antioxidant phenolics can also be a response of this plant to water suppression (caffeate × RWC, *r* = −0.67, *P* = 0.06). To our knowledge, this was the first study that reports the presence of caffeoylquinic acid in leaves of resurrection plant species. Therefore, our findings suggest that the metabolome of *B. pupurea* related to phenolic acids in the summer was ready to cope with dehydration due to a priming caused by an increase of light and temperature.

*B. purpurea* occurs in monocotyledons mats on soil islands, some meters above the tidal zone, where it is exposed to great temperature variations, frequent periods without precipitation and to salinity effects (Meirelles et al., 1999; Aidar et al., 2010). Generation of ROS is a common stress-induced response in plants and accumulation of antioxidant phenolics in *B. purpurea* during the summer experiment, although triggered by other environmental factors, certainly also contributes to its tolerance desiccation.

The synthesis of amino acid hydroxyproline, as well as the increase of serine, valine, histidine, and tyrosine can be an indicative of deposition of extensins in cell wall of *B. purpurea*. Extensins are classified as members of a family of glycoproteins enriched in hydroxyproline, which provide stability to cell wall (Cassab, 1998). The alterations in the levels of hydroxyproline may be associated with an increase in hardness of leaves of *B. purpurea* observed upon drought stress conditions, providing mechanical resistance against the cell wall stress caused by drought.

The increase of amino acids occurred in RWC below 70% suggesting a decrease in the carbon/nitrogen ratio, resulting in increase in the efficiency of transport of nitrogen to the aerial part of the plant. The accumulation of amino acids in the initial phase of drought and its maintenance during stress indicates a cellular osmotic adjustment for maintaining the leaf turgidity as an early plant response under these conditions. In beet plants, Gzik (1996) observed that increased levels of amino acids were related to osmotic adjustment for stabilization of water state in tissues under water deficit conditions. Also, the dehydration could be stimulating the metabolism deviation for other routes. The increase in tryptophan and decrease in shikimic acid observed in *B. purpurea* suggest a change in metabolism toward the secondary metabolite production, which develops an important role on desiccation tolerance.

Other functions of the amino acids in metabolic maintenance under stress conditions were related. The accumulation of asparagine in vegetative tissues occurs in response to saline and water stress, being related to a restriction of protein synthesis rates, and may still be associated to osmotic regulation (Lea et al., 2006). Moreover, the accumulation of GABA occurs in response to various environmental stresses (Shelp et al., 1999; Akçay et al., 2012). Accumulation of amino acids can be also associated to storage of available substrate for protein synthesis and quick recover of the plant metabolism after rehydration.

A holistic view of the metabolism reported by the PCA analysis based on the MP (Figure 7) indicates that in *B. purpurea* the biochemical adjustments start early in the drying, preparing the plants for a period of stress. Interestingly, it seems that changes in winter were more pronounced than those observed in the summer experiments. Based on this fact we could speculate that during summer plants are prepared for more drastic decrease in water availability at any time due to high temperatures and irradiance.

### Rehydration

After rehydration we observed that recovery of leaf turgor in *B. pupurea* occurred from the base to the leaf apex, slowly and apparently programmed, leading to unfolding of the leaf tissues and with a gradually green color return.

The contents of sucrose and RFO accumulated during dehydration decreased after rehydration of the pots, in both winter and summer at RWC above 80%. These decreases indicate, probably, that the carbohydrates were used as a carbon source in energetic-dependent metabolic pathways responsible for repairing the damages caused by desiccation, as suggested by Peters et al. (2007) for *X. viscosa*. This is possible because these carbohydrates, weak reducing agents, can act as a carbon stock (Toldi et al., 2009). The levels of amino acids accumulated also decreased after rehydration, indicating that nitrogen accumulated may has been used as substrate for protein replacement (Shelp et al., 1999). This strategy seems to be slightly different from that used by other species. In *H. rhodopensis*, Gechev et al. (2013) observed a decrease for some of the analyzed amino acids, such as aspartate and asparagine, with posterior increase to control levels after rehydration, in opposite direction of those found in *B. purpurea*. On the other hand, phenylalanine also increased during dehydration and decreased to control levels after rehydration, indicating that carbon input on secondary metabolism is not priority at high water availability for both species.

Similarly, GABA, which increased during dehydration in *B. purpurea* returned to control levels after water deficit suspension. GABA is known to respond to different environmental stress (Akçay et al., 2012) and acts as a signaling factor. Renault et al. (2011) reported that GABA accumulation affects cell-wall elongation in both reproductive and vegetative tissues. Moreover, accumulation of GABA in *Arabidopsis* roots resulted in accumulation of amino acids and decrease in carbohydrates, affecting the central C/N metabolism, by changing GABA transaminase (Renault et al., 2010). In agreement to these findings, our metabolite measurements in *B. purpurea* showed the increase in the amino acid content, such as asparagine, which is more efficient in nitrogen transport from the roots to the shoots. After rehydration, the levels of these metabolites decreased since the more efficient nitrogen transport was no longer required.

With rehydration, we observed all metabolic alterations that involve the maintenance of cell viability in the dried in *B. purpurea* returning to normal conditions until 132 h after resumption of irrigation. Intriguingly, the metabolism after rehydration in winter experiment returns to its basal levels faster than summer experiments, which can be observed when the PCA is analyzed (Figure 7). This could be also associated to the different environmental conditions, which, even under high water availability is still submitted to factors of stress, such as temperature and light intensity, during the summer. This time of recovery seems to be necessary for regulation of metabolism and may be associated with the repair of possible damage at this stage, as suggested by other studies on resurrection plants (Oliver et al., 1998; Farrant et al., 2007; Lüttge et al., 2008; Farrant et al., 2012). Recently, Velitchkova et al. (2013) demonstrated that high temperature has stronger physiological effects on the dehydrated plants of *H. rhodopensis*, a resurrection species of the northern hemisphere, reducing its capacity of recovering after rehydration, when compared to plants submitted to desiccation at optimal temperature.

## Conclusions

Metabolic changes in *B. purpurea* are associated with a time-dependent response as a function of the intensity of the water deficit in summer season experimented by plants in greenhouse. Data from summer experiment suggest that the metabolism of *B. pupurea* was already primed for drought stress prior the water deprivation, as observed by the constitute amounts of caffeoyl quinic acid derivatives and higher accumulation of verbascose.

**Figure 8:**
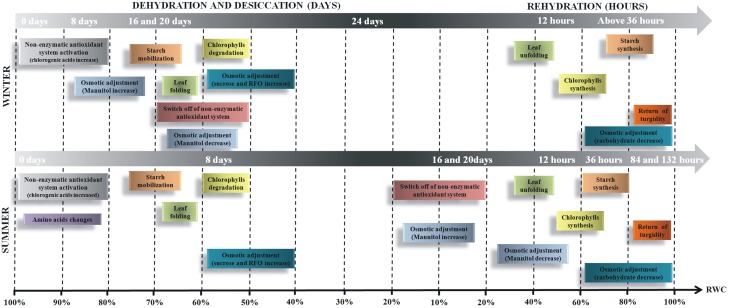
**Schematic representation of changes in metabolism during dehydration and rehydration in *Barbacenia purpurea* plants**. The time-dependent events observed in *B. purpurea* plants cultivated and submitted to extreme water deficit during winter and summer season.

Tissue water content seems to be the major signal for desiccation tolerance in this species in the winter season. Thus, from all data compiled, we verified that the responses are time-dependent and may be divided in two steps (Figure 8). The first responses occur in RWC between 96 and 70% and are characterized by activation of an antioxidant defense system, supported by the increased levels of quinic acid and caffeoylquinic acids. It is followed by osmotic adjustment supported by the increased levels of some amino acids associated to mechanisms to avoid desiccation as a delay in leaf dehydration. At RWC below 70%, the results indicate that carbohydrates play a crucial role in the osmotic adjustment besides acting on the protection of cellular components, such as membranes and proteins. Carbohydrates and amino acids accumulated at the final stage of drying seem to be important sources of carbon and nitrogen for use in the subsequent rehydration. Our findings support the definition of desiccation tolerance in vascular homeohydrics, which are characterized by activation of avoidance of dehydration associated with protection mechanisms in drying and repair on rehydration.

## Author contributions

Vanessa F. Suguiyama got her MSc. degree working on this subject and was responsible for performing all experiments. Emerson A. Silva is expert in water relations of plants and soils and helped with the experimental design and ecophysiological measurements. Sergio T. Meirelles provided the plants of *B. pupurea* and helped with his expertise in cultivating and imposing the water deficit in the species. Danilo C. Centeno is expert in metabolomics and helped with the GC/MS analyses and interpretation of the results. Marcia R. Braga is expert in plant carbohydrates and the group leader, being responsible for the supervision of the MSc. project of Vanessa F. Suguiyama. All co-authors contributed to the paper writing and discussing the results.

### Conflict of interest statement

The authors declare that the research was conducted in the absence of any commercial or financial relationships that could be construed as a potential conflict of interest.

## Abbreviations

GC/MS: gas chromatography coupled to mass spectrometry
GABA: gamma-aminobutyric acid
HPLC: high performance liquid chromatography
HPAEC/PAD: high performance anion exchange chromatography coupled to pulse amperometric detector
MP: metabolite profile
PCA: principal component analysis
RFO: raffinose-family oligosaccharides
RH: relative humidity
ROS: reactive oxygen species
RWC: relative water content
UHPLC: ultra performance liquid chromatography.
